# Auditory salience using natural scenes: An online study

**DOI:** 10.1121/10.0006750

**Published:** 2021-10-19

**Authors:** Sandeep Reddy Kothinti, Nicholas Huang, Mounya Elhilali

**Affiliations:** 1 Department of Electrical and Computer Engineering, Center for Language and Speech Processing, The Johns Hopkins University, Baltimore, Maryland 21218, USA; 2 Department of Biomedical Engineering, The Johns Hopkins University, Baltimore, Maryland 21218, USA

## Abstract

Salience is the quality of a sensory signal that attracts involuntary attention in humans. While it primarily reflects conspicuous physical attributes of a scene, our understanding of processes underlying what makes a certain object or event salient remains limited. In the vision literature, experimental results, theoretical accounts, and large amounts of eye-tracking data using rich stimuli have shed light on some of the underpinnings of visual salience in the brain. In contrast, studies of auditory salience have lagged behind due to limitations in both experimental designs and stimulus datasets used to probe the question of salience in complex everyday soundscapes. In this work, we deploy an online platform to study salience using a dichotic listening paradigm with natural auditory stimuli. The study validates crowd-sourcing as a reliable platform to collect behavioral responses to auditory salience by comparing experimental outcomes to findings acquired in a controlled laboratory setting. A model-based analysis demonstrates the benefits of extending behavioral measures of salience to broader selection of auditory scenes and larger pools of subjects. Overall, this effort extends our current knowledge of auditory salience in everyday soundscapes and highlights the limitations of low-level acoustic attributes in capturing the richness of natural soundscapes.

## I. INTRODUCTION

In everyday life, a multitude of information-bearing sources impinges on our senses that it is almost impossible to process all the information at once with the same resolution. Attention plays a vital role in focusing perceptual and cognitive resources in navigating the real-world (Driver, 2001). Attention can be employed voluntarily to perform a task efficiently, such as paying attention to a particular individual at a cocktail party (Cherry, 1953). This is an example of top-down attention, where a listener's goal orients her perceptual and cognitive resources to facilitate listening to the desired signal amidst other distractors (Baluch and Itti, 2011). In contrast, a source or stimulus can also capture our attention because of its inherent properties, known as salience; for instance, the sound of glass shattering, if someone drops a glass at the same party, would be a salient sound that attracts our attention regardless of our state of cognitive control or attentional focus. Salience presents itself in various perceptual modalities; a flashing traffic sign on the road (visual), a brewing smell of coffee (olfaction), and the earlier example of glass breaking (auditory) are all salient but in different ways. Understanding salience mechanisms provide insights not only into perceptual and cognitive systems in the brain but also guides the development of technologies that can more efficiently process information in real-life scenarios.

The literature on sensory salience has varied greatly, particularly in terms of appropriate experimental paradigms best suited to shed light on underlying physical, neural, and perceptual underpinnings of salience encoding in the brain. In visual salience studies, gaze-tracking paradigms have become a standard approach to track the eye movements of a subject when presented with a static image or video in free viewing tasks. In the absence of any task demands, the tendency to fixate gaze on specific locations is guided by image features which inform of salience attributes of both low-level and high-level visual information in the image (Bruce and Tsotsos, 2009; Judd *et al.*, 2009; Zhao and Koch, 2013). In contrast, in the auditory domain, there is no established framework to study salience (Kaya and Elhilali, 2017). Some experimental schemes examine auditory salience as the ability of the stimulus to pop-out while the subject is actively engaged in the task (Kaya *et al.*, 2020; Southwell *et al.*, 2017), whereas others use attention tracking mechanisms to measure salience continuously while subjects are attending to the stimuli (Huang and Elhilali, 2017; Zhao *et al.*, 2019b). In addition, distractor paradigms are considered in some studies to probe attentional deployment in the presence of distracting salient events (Petsas *et al.*, 2016; Southwell *et al.*, 2017; Vachon *et al.*, 2017); alternatively, detection tasks collect feedback from subjects about their judgments of event salience or relative salience after a stimulus is presented (Kaya and Elhilali, 2014; Kayser *et al.*, 2005). Ultimately, these tasks yield different outcomes in terms of discrete versus continuous measures of salience over the course of an entire acoustic signal. They also probe various aspects related to auditory salience as it pertains to either the encoding of specific events or entire scenes or its effect on attentional deployment and perceptual or cognitive load necessary to perform the specific task at hand. Given that sounds unfold over time, it is advantageous to obtain a continuous temporal measure of salience as humans interact with complex soundscapes with dynamic attentional guidance based on the attributes of the sound events in the scene.

In addition, measures of salience are heavily informed and sometimes biased towards the choice of stimuli used in the experiments. Early visual salience studies used abstract shapes such as lines and polygons to identify effects of similarity and differences in simple attributes like colors and shapes (Treisman and Gelade, 1980). Salience models developed based on these simplified stimuli often failed to generalize to complex natural scenes (Itti and Koch, 2000; Judd *et al.*, 2009). In recent years, visual salience has been more commonly explored using complex natural scenes such as faces (Ramanathan *et al.*, 2010), natural images (Judd *et al.*, 2009), and images with rich contextual information (Jiang *et al.*, 2015); hence resulting in richer and more generalizable models and theories of visual salience. In the auditory domain, synthetic or simulated data such as sequences of tones (Duangudom and Anderson, 2013) or odd-ball musical sequences (Kaya and Elhilali, 2014) were used to identify salience effects along known auditory attributes, such as loudness, pitch, and timbre. Similarly, natural auditory stimuli present more complexity when compared to simulated data and require a higher level of control on the biases introduced by familiarity, semantic information, as well as sound context. Moreover, natural stimuli present an additional challenge in terms of large variations or configurations in which certain aspects of the scene can be presented. To eliminate some of these confounding factors when probing general effects of auditory salience, it is important to explore a large variety of natural scenes.

The experimental design and complexity of incorporating richer stimuli also raise challenges with regard to the number of subjects and variability arising from pool selection (de Haas *et al.*, 2019). Conventionally, salience experiments are conducted in laboratory settings, which offer control over the testing conditions and the quality of the subject responses. However, laboratory experiments are time-consuming and often lack subject diversity due to limitations on both subject pool and size, which can bias the study findings. In recent years, online platforms have emerged as a medium for large-scale data collection, allowing data acquisition and curation to grow in orders of magnitude [e.g., 14 × 10^6^ annotated images in ImageNet (Deng *et al.*, 2009), 5.8 thousand hours of audio in AudioSet (Gemmeke *et al.*, 2017)]. Crowd-sourcing has also been leveraged more recently in several behavioral and psychophysical studies to not only overcome challenges with performing such tests in the laboratory, but also to broaden access to a larger pool of volunteers and cover more diversity in age, race, and gender (Buhrmester *et al.*, 2011; Buhrmester *et al.*, 2018). Nonetheless, relinquishing control and rigor of an experimental setup in the lab under the scrutiny of the researcher comes at the cost of questionable quality and interpretability of the crowd-sourced results. Despite the skepticism regarding online data collection, visual salience studies have embraced crowd-sourcing as the *defacto* platform for large-scale data collection over the years. In visual salience studies, eye-tracking and visual search paradigms are prevalent paradigms. While visual search paradigms measure salience as an attribute of a target object in the image, eye-tracking-based paradigms employ *free-viewing* (Borji *et al.*, 2013; Zhao and Koch, 2013) and track eye-movement while the subject processes the image. Eye-tracking paradigms provide a salience map of each image and can be used to study natural images. Crowd-sourcing has been extensively used to collect eye-tracking data by using the web camera (webcam) at the side of the subject (Huang *et al.*, 2019; Kim *et al.*, 2015; Xu *et al.*, 2015) or mouse-contingent paradigms (Gomez *et al.*, 2017; Lyudvichenko and Vatolin, 2019; Newman *et al.*, 2020), which emulate human visual exploration by tracking the mouse movement. Several studies have compared eye-tracking data on datasets collected in laboratory environments with crowd-sourced data and reported that crowd-sourced data match closely with traditional data collection paradigms (Jiang *et al.*, 2015; Rudoy *et al.*, 2012). This consistency has led to the deployment of crowd-sourcing for large-scale salience data collection. SALICON (Jiang *et al.*, 2015) is an eye-tracking dataset collected using a mouse-contingent paradigm for 10 000 images and is presently the largest salience dataset. Several deep learning models have been successfully trained on this dataset (Borji, 2021; Cornia *et al.*, 2018; Kruthiventi *et al.*, 2017). By establishing crowd-sourcing as a reliable framework for data collection, visual salience datasets have not only exponentially grown in size, but also opened new avenues to developing improved models for various downstream tasks including objective video quality assessment (Le Meur *et al.*, 2007) and region of interest (ROI) identification in video (Rai *et al.*, 2016).

In comparison, scaling up auditory salience datasets remains in its infancy. A recent salience study adopted crowd-sourcing for salience judgment tasks (Zhao *et al.*, 2019a) and showed a strong agreement between the laboratory data and the crowd-sourced data. Still, several hurdles remain in terms of scaling up the study of auditory salience, both in terms of scope, size, and diversity of stimuli, as well as the choice of paradigms that—at the very least—yield temporal salience maps that can be leveraged to tie in multi-scale representations of natural sounds (from low-level acoustics to high-level semantics). It is therefore important to scale studies of auditory salience to not only broaden the scope and diversity of stimuli but also examine effects across larger pools of subjects.

The present study aims to provide insight into adopting crowd-sourcing as the primary experimental platform for auditory salience experiments. For this purpose, we adopt the dichotic listening task used in an earlier study (Huang and Elhilali, 2017) for a web-based crowd-sourcing platform. The study sets out three main goals. First, it evaluates the use of a crowd-sourcing platform to yield high-quality salience measures using a dichotic listening paradigm. Salience data collected in the laboratory in the previous study [JHU-DNSS (Dichotic Natural Salience Soundscapes)] is compared with data collected using the popular crowd-sourcing platform Amazon Mechanical Turk (MTurk). To establish consistency, a cross-platform comparison is performed on responses from the two settings, as well as salience models derived from the two platforms. Second, it extends the selection of stimuli used previously (JHU-DNSS) to encompass a wider collection of event types and environments; particularly focusing on acoustically dense scenes that cause more challenges of interpretability and predictability for salience models. Third, it evaluates salience responses derived from a larger and diverse pool of subjects using data-driven salience models. The effect of larger subject size on training and evaluating salience models is assessed. Ultimately, the study aims to expand the frontiers of auditory salience using larger datasets of complex sounds.

## II. METHODS

### A. Behavioral procedure

*Experimental setup:* Behavioral data from an online platform were collected using Amazon Mechanical Turk (MTurk). The experiment was conducted using a web server hosted on Amazon Web Services (AWS), and the interface with AWS was enabled by the psiTurk framework (Gureckis *et al.*, 2016), which uploaded the experiment as an MTurk Human Intelligence Task (HIT). Once the task was posted, subjects were able to find the HIT, complete the task, and receive payment without further interaction with the experimenter. The experiment presentation was implemented using the jsPsych library (de Leeuw, 2015). Crowd-sourced participants were instructed to use headphones for the task. Subjects were instructed to adjust the volume to a comfortable level before beginning the experiment. A training phase was conducted to familiarize subjects with the interface, check if the orientation of the headphones was correct, and confirm that dichotic listening was achieved. An optional break of 30 s was provided between trials. This crowd-sourced data (referred to as *crowd*) was compared against data previously collected in a booth in a laboratory setting (referred to as *booth*), with subjects seated in a soundproof booth and audio presented over Sennheiser HD595 headphones, under close supervision by an experimenter (Huang and Elhilali, 2017).

*Auditory stimuli:* In total, 56 audio recordings of natural scenes with a total duration of 112 min were used as stimuli for behavioral data collection. These scenes included 16 scenes from the JHU-DNSS database previously used to collect *booth* data. An additional 40 scenes (DNSS-Ext) were selected to extend the DNSS dataset, both to a larger size and greater variety of scenes. Scenes were chosen specifically to include more events of underrepresented classes in the original database (e.g., animal sounds and non-speech human vocalizations). In addition, scenes were chosen to include mostly acoustically dense scenes, i.e., scenes with continuous presence of one or more overlapping auditory objects and fewer quieter moments. Those scenes were found to have events that were difficult to predict from acoustic models (Huang and Elhilali, 2017). All the 40 new scenes were exactly two minutes in length and were taken from the Freesound audio library (Freesound, 2021). All auditory stimuli were sampled or resampled at 22 kHz with 32 bits per sample. Any stereo recordings were converted to mono by taking the average of the two channels.

For data collection purposes, auditory stimuli were divided into three blocks with 16 scenes from the original study as one block (DNSS) and two subsets of 20 scenes from the DNSS-Ext stimuli as two separate blocks. Table I summarizes the scene breakup for DNSS and DNSS-Ext blocks. More detailed description of the scenes is provided in the Appendix. Scenes were subjectively labeled by the experimenter as dense if there were one or more object present throughout the scene and as sparse if there are multiple segments of the audio without any acoustic energy.

**TABLE I.** Description and grouping of the scenes used in the study.

Set	# Blocks	# Scenes	Duration	# Dense
DNSS	1	16	1.13–2.22	10
DNSS-Ext	2	40	2.00	40

*Behavioral paradigm:* Following the same procedure adopted to collect *booth* data [see Huang and Elhilali (2017)], online participants were presented with two auditory scenes dichotically. Subjects listened concurrently to both scenes, one delivered to each ear, and indicated their attentional focus continuously by positioning their cursor on the screen. They were instructed to move their cursor to the right side of the screen when the scene played in the right ear grabbed their attention, and vice versa when attending to the scene in their left ear. When attending to both scenes or neither of them, subjects were instructed to keep their cursor in the center of the screen. Participants were instructed to move the cursor as soon as they noticed that their attention had shifted, and vertical lines divided the screens into sections to delineate the three response areas (left, right, and middle).

**FIG. 1.**
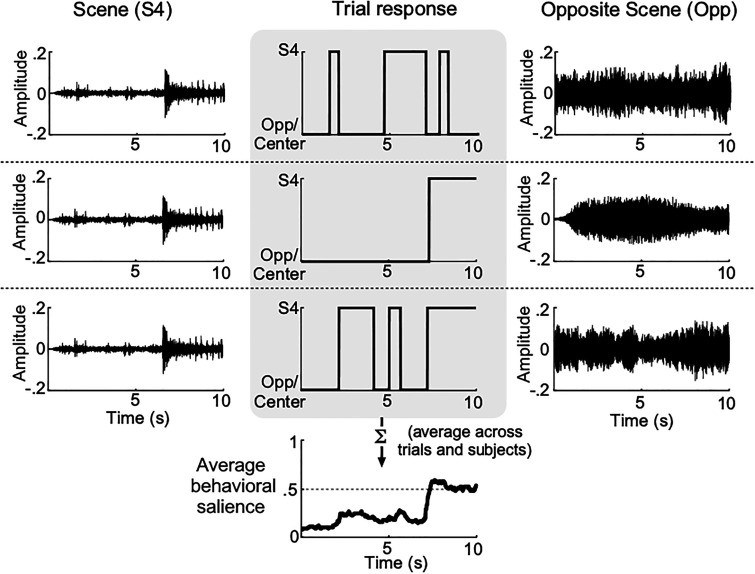
Salience measurement paradigm. Two scenes were dichotically presented, and subjects indicated continuously which scene they were attending to at any given time. Three example trials are shown in the figure with the same scene (scene 4) being paired with different opposing scenes. The middle panel shows responses for the example trials. Responses were averaged across subjects to achieve a salience measure called average behavioral salience as shown in the bottom-most panel.

Each scene was paired with different opposing scenes across subjects and trials, and the responses from all the trials were averaged to get the mean salience measure of the scene over time across subjects and opposing scenes (Fig. 1). Each experiment consisted of 10 trials, during which two scenes from a block of scenes were chosen randomly without replacement which ensured that a subject listens to each scene only once. Experiments were designed such that each scene is paired on average with 18 different scenes, and a specific pair of scenes repeated once in every 18 subjects on average. This procedure aligns with the procedure used for *booth* data. Subjects spent 30 min of experimental time on average excluding the breaks in both laboratory and crowd-sourced settings.

*Participants:* In this study, a total of 275 subjects (154 male, 110 female, 11 non-binary/unspecified) with average age of 36.4 years (standard deviation = 12.8 years) were recruited as part of *crowd* data. Subjects were asked to report the languages they speak, their dominant hand, and whether they have normal hearing. Subjects were compensated for the task after data collection following a study protocol approved by the Johns Hopkins Institutional Review Board (IRB).

Subjects for data collection in the sound-booth were recruited on the university campus. *Booth* data consisted of responses from 50 subjects (16 male, 34 female) with average age of 21.8 years (std dev = 3.8 years). Subject populations in *booth* were significantly younger and comprised of higher percentage of female participants when compared to *crowd*.

### B. Behavioral data analysis

*Data quality control:* Subject responses in both *crowd* and *booth* data were analyzed based on the speed of cursor movement or switching rate across the three positions in the screen (left, right, middle). The average rate of attentional switches (a shift of attention from one scene to the other) was used as a criterion to flag trials with outlier behavior. Trials with disproportionately large (above 1/s) or small (0.025/s) switches were considered outliers and removed from further analysis. Subjects with more than half of the trials flagged as anomalous were identified as outlier subjects and were excluded from further data analysis.

*Defining salient events*: A continuous salience measure was defined as the fraction of subjects listening to a given scene at any given time, averaged across many subjects and competing scenes, as shown in Fig. 1, bottom middle panel. For each scene in a trial, subject responses were mapped to 1 for subjects listening to the scene and 0 for subjects listening to the opposite scene or neither scenes nor both scenes (quantitatively and qualitatively similar results were obtained if responses to the center of the screen and opposite scene were differentiated). An average response curve called average behavioral salience was obtained by taking the average of subject responses for each scene.

Onsets of salient events were defined as peaks in the slope of the average behavioral salience curve. These events were moments in the scenes when a large number of subjects began attending to that particular scene, regardless of the contents of any opposing scene. To identify peak locations, the salience curve for each scene was first smoothed with three equally weighted moving average operations of duration 1.5 s. Then a peak-detection method was applied to find timestamps of local maxima in the first-order difference of the smoothed salience curve. A fixed duration of 1 s was subtracted from the peak timestamps to obtain salient event onsets. This adjustment was done to account for subject reaction times. 1 s was found to be a typical time difference between a peak in loudness change and the next salient onset in both *booth* and *crowd* datasets [see Huang and Elhilali (2017) for details].

The strength of each event was computed as a sum of two factors: (1) height of the local maximum of the 1st order derivative of salience, which measures temporal agreement among subjects, and (2) maximum salience within 4 s after the event scaled by 75th percentile of slopes, which reflects absolute consensus irrespective of time. Events identified using this procedure were sorted based on salience strength, and the top 50% events were chosen as the final events. This additional pruning was performed to remove small peaks that can arise because of noise in behavioral responses.

*Reaction times:* Reaction times were computed for each subject by comparing individual subject responses to the onset of salient events (event onsets are defined as the time when the “average subject” responded with 1 s subtracted for nominal reaction time). A subject was considered to have responded to a salient event if they moved towards the particular scene between 0.5 and 1.5 s after the event onset. The difference between the event timestamp and the time when the individual subject moved the mouse towards the scene was considered as the event reaction time.

*Behavioral response consistency*: A subset of the *crowd* data (only matching DNSS stimuli) was compared to *booth* data, to check for consistency across platforms on the same stimuli using two metrics: correlation and subject-wise F-scores. (1) Correlations were defined for individual scenes. These were computed as sample Pearson correlation between the average behavioral salience of matched scenes from *crowd* and *booth* by assuming average behavioral salience at different time-points as samples; then correlation coefficients were averaged across scenes. (2) F-scores were computed for individual subjects. Instances, when the subject moved towards a given scene, were identified as events detected by that subject; reference salient events were derived from the average behavioral salience of the remaining subjects for that scene (following the procedure to derive salient events described earlier). A detection analysis was performed to evaluate the subject's detected events against reference events. Matched detections (within 1 s) were considered as hits. Reference events without a close-by detected event (within 1 s) were considered false negatives, and detected events without any close-by reference event were considered false positives. F-score was computed as a harmonic mean of precision and recall with appropriate flooring (Mesaros *et al.*, 2010). These two metrics were chosen because they reflect different aspects of consistency across platforms: The correlation metric indicated how well the average behavioral salience profiles from *booth* and *crowd* match; while subject-wise F-scores indicated how well a subject agreed with the average behavioral salience around salient moments in a scene and hence reflected a level of uniformity in subjects' responses in both platforms.

*Effect of subject size:* The number of online subjects recruited for the study can affect variability in the data. The interobserver agreement on events from *crowd* was evaluated as a function of number of subjects. It quantified how well subjects agreed with salient events derived from the average behavioral salience of all the subjects in *crowd* data. This analysis was performed on the DNSS scenes only since these scenes were tested in both *booth* and *crowd* settings. To evaluate interobserver agreement, scenes were divided into segments of 1 s with 75% overlap, and each segment was labeled 1 or 0 based on whether a salient event was present in the segment or not, respectively. The percentage of subjects who switched their attention within each segment was used as the detection signal. Hits and false alarms were computed with different threshold levels. The area under the ROC curve (AUROC) (Fawcett, 2006) was computed as a concise metric of detection performance. A similar analysis was performed on the *booth* data using the entire booth subject pool and used as benchmark for the *crowd* data. Along with the *booth* interobserver AUROC, a chance-level benchmark was computed by shuffling the dataset to form randomized datasets. For each scene, responses from the *crowd* data were sampled without replacement from the pool of responses from all subjects and scenes. Interobserver agreement in terms of AUROC was computed for the shuffled dataset by finding salient events from the average behavioral salience computed from the shuffled dataset and performing the detection analysis from the individual responses as described above. This procedure was repeated 50 times each for various subsample sizes. The AUROC values produced in this way indicated a chance level interobserver agreement.

### C. Acoustic analysis

*Feature analysis*: A set of eleven acoustic features that capture a wide range of spectral, temporal, and spectro-temporal attributes of auditory scenes were used to analyze acoustic markers of auditory salience. All spectral features were computed by first converting the audio waveform *x*(*t*) to an auditory spectrogram *y*(*t*, *f*) using the biomimetic model of Wang and Shamma (1994) using a frame rate of 125 Hz.

(1) Loudness (LD) was derived by taking the average of envelopes computed on 28 bark frequency bands (Zwicker *et al.*, 1991) in the range [250 Hz, 12 kHz] with critical bands matching mammalian auditory periphery.
(2) Pitch (P) was computed using the optimum processor method (Goldstein, 1973), where each spectral slice was matched with a set of pre-computed pitch templates and the fundamental frequency was determined from the best template using maximum likelihood estimation.
(3) Harmonicity (H) was derived as a measure of the degree of match between each spectral slice and the matched pitch template.
(4) Brightness (BR), or spectral centroid, was computed as the weighted average of frequencies with the power spectrum at each time [ $y{\left(t,f\right)}^{2}$] as weights.
(5) Bandwidth (BW) was computed as an average of the absolute difference between the spectral centroid and frequencies, weighted by the magnitude spectrum.
(6) Irregularity (IR) measures jaggedness in the spectrum and was computed as a sum of squares of the difference between consecutive spectral magnitudes divided by the sum of squares of spectral magnitudes.
(7) Flatness (FL) was computed as a ratio of geometric mean to the arithmetic mean of spectral magnitudes.
(8) Average slow temporal modulations (or low rates -LR) were computed using the NSL toolbox (Chi *et al.*, 2005) by decomposing spectrogram *y*(*t*, *f*) into temporal modulations *z*(*t*, *r*) for r∈[1,20] Hz, then averaging the energy across modulation filter outputs.
(9) Average fast temporal modulations [or high rates (HR)] were computed similarly as above, by averaging energy in temporal filters r∈[20,100] Hz. This average energy is commensurate with a measure of roughness previously found to correlate with aversion to auditory events (Arnal *et al.*, 2019).
(10) Rate centroid (RC) was computed as the centroid of temporal modulations over the range r∈[1,32] Hz.
(11) Scale centroid (SC) was computed similarly to rate by first decomposing the spectrogram *y*(*t*, *f*) into spectral modulations *w*(*t*, *s*) for s∈[0.25,8] cyc/oct and then taking the centroid.

These features were chosen to span a wide range of potentially relevant acoustic attributes, including many that have been found to be predictors of salience in prior studies. Temporal and spectral modulations were part of the auditory salience map model by Kayser *et al.* (2005). Contrasts in loudness and pitch were demonstrated to be salient by Kaya and Elhilali (2014), and Tordini *et al.* (2015) found that less bright sounds were more likely to be in the foreground. While the remaining features were not studied in the context of auditory salience prior to the original study, they were recognized to be useful for various audio applications (Alías *et al.*, 2016).

All features were resampled to have a uniform sampling time of 64 ms. They were normalized to have zero mean and unit variance. To analyze the dependencies across these features, Pearson correlations were computed for DNSS and DNSS-Ext scenes for each pair of features. Changes in features around events were analyzed to appraise the contribution of specific acoustic features in the event salience. The change in an acoustic feature was computed as the difference between the average value of the feature in two 0.5 s intervals: one starting at 0.5 s after event onset and one starting 1 s before event onset.

*Acoustic prediction of events:* A model to predict salient events was developed based on the acoustic analysis. The model operates in a segment-wise manner where each scene was first split into segments of 1 s with an overlap of 0.75 s, and each segment was assigned a label 1 if an event was present in the segment or 0 if no event was present in the segment. A change detection based approach was used on the acoustic features for event prediction. For each feature, peaks in the slope of the feature were assumed to be predicted events for the feature-derived salient events. Events predicted across features were converted to segment-level binary predictions and combined using the linear discriminant analysis (LDA) method (Fisher, 1935). The LDA weights signified how individual acoustic features contributed to predictions of salience of the scene. Segment-level salient event labels were used as class assignments, and feature-based predictions were used as inputs to the LDA procedure. LDA model parameters were trained and tested with non-overlapping subsets of the data using 10-fold cross-validation. During an evaluation with the held-out set, LDA output was binarized with different thresholds and compared with the labels to compute hits and false alarms at each threshold level. Area under the receiver operating characteristic curve (AUROC) was used as a summary statistic to compare different LDA model performances. While the detection methodology has similarities to sound event detection [e.g., DCASE 2017 (Mesaros *et al.*, 2019)], the formulation in this work predicts the onsets derived from behavioral salience as opposed to just acoustics. This methodology was adopted to highlight how the acoustic dimensions were driving the salience by using a data-driven approach.

*Data augmentation:* Two different sets of LDA models were trained using *booth* and *crowd* data for DNSS scenes and were evaluated with held-out sets from both the platforms to examine the compatibility of LDA models from one platform to the other. To understand the benefits of larger data, additional models were trained using varying amounts of added training data and again evaluated on held-out sets. For this analysis, *booth* and *crowd* subject data for matched DNSS scenes were combined. The total data were randomly sampled 100 times for each training data size to capture variations in the data.

## III. RESULTS

### A. Behavioral results

**FIG. 2.**
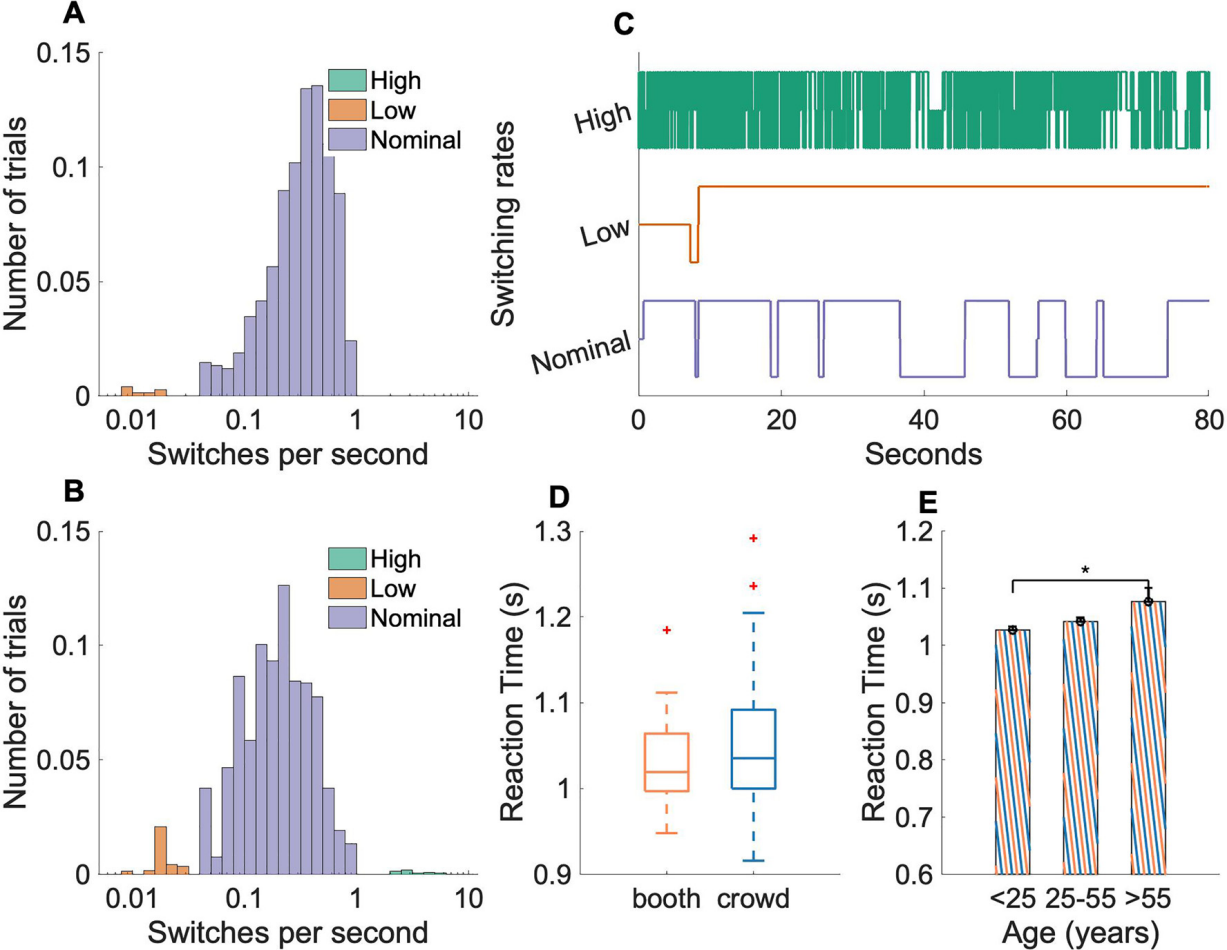
(Color online) (A) Distribution of the number of switches across trials in the sound booth data. 4% of trials had a low switch rate, while 0% of trials had a high switch rate. (B) Distribution of the number of switches across trials in the Mechanical Turk data. 4% of trials had a low switch rate while 2% of trials had a high switch rate. (C) Example responses that exhibited low, regular, and high numbers of attentional switches between scenes. Trials with low or high switches were excluded from the analysis. (D) Box plot of subject reaction times with median, 25th and 75th percentiles for DNSS scenes from *booth* and *crowd* data. (E) Reaction times from combined *booth* and *crowd* data divided by subject age. Error bars represent ±1 standard error and significant differences are indicated by stars.

Analysis of behavioral responses across subjects comparing *booth* and *crowd* data reveals slightly different patterns of switching rate before any outlier analysis [Fig. 2(A) and 2(B)]. The median switching rate of *booth* (0.32 switches per second) was found to be higher than *crowd* (0.17 switches per second) [Wilcoxon rank-sum test (Wilcoxon, 1945), *p* =  2e − 61]. Trials with disproportionately large [Fig. 2(C), top] or small [Fig. 2(C), middle] number of attentional switches were excluded from further analysis. Within the *crowd* data, roughly 6% of trials (upper 2 percentile, lower 4 percentile) had either a high or a low rate of attentional switches. Overall, 12 of 275 subjects were identified as outlier subjects and all the trials from these subjects were removed. Removing outlier trials increased the median switching rate in *crowd* to 0.19 switches per second. For *booth* data, no subjects were considered as outliers as their switching patterns remained largely within the nominal switching rates.

We also analyzed average reaction times under both experimental paradigms. Figure 2(D) shows reaction time distributions for *booth* and *crowd* with median, 25th and 75th percentiles for the DNSS scenes. Reaction times from both paradigms followed a normal distribution [Kolmogorov-Smirnov test (Massey, 1951), *p*(*booth*) = 0.64, *p*(*crowd*) = 0.47] and were not significantly different from each other [two sample t-test, *t*(138) = –1.69, *p =* 0.09].

To further explore similarities and differences between *booth* and *crowd* data, a three-way ANOVA on reaction time was performed using age (<25 and ≥ 25), platform (*booth* and *crowd*), and gender (male and female). Subjects without gender information (four subjects who listened to the DNSS scenes only) were removed for this analysis. As shown in Table II, none of the factors or interactions were found to be statistically significant. No effect of platform indicated that both paradigms had subject responses with similar latencies. The effect of age and interaction between age and platform were found to be contributing the most to the differences in reaction times than other factors. These age effects (and interaction between age and platform) were primarily caused by imbalances in subject ages in the two paradigms with most subjects from *booth* being younger (92% of subjects with age <25) than *crowd* (12% of subjects with age <25). To understand the effect of age further, subjects from *booth* and *crowd* were grouped based on subject age to balance the age distributions more evenly [Fig. 2(D)]. There were no differences between the young-age (<25) and middle-age ( ≥25 and ≤55) groups [two-sample t-test, t(128)=−1.51, *p =* 0.09], and the middle-age and old-age (>55) groups [two-sample t-test, t(88)=−1.56, *p =* 0.12]; however, the old-age group had slower reaction times [one-sided t-test, t(58)=−2.62, *p =* 0.015] compared to the younger group.

**TABLE II.** ANOVA results for subject-wise F-scores and reaction times. Three factors considered were Age (>25, ≤25), Gender (male, female), and Platform (*booth*, *crowd*).

		Reaction times			F-scores		
Factor	df	Mean. Sq.	F	p	Mean. Sq.	F	p
Age	1	0.0076	2.28	0.13	0.0009	0.23	0.63
Platform	1	0.0006	0.19	0.66	0.0000	0.01	0.93
Gender	1	0.0000	0	0.99	0.0079	1.94	0.17
Age × Platform	1	0.0079	2.35	0.13	0.0000	0.02	0.90
Age × Gender	1	0.0001	0.05	0.83	0.0008	0.19	0.66
Platform × Gender	1	0.0008	0.24	0.63	0.003	0.76	0.38

**FIG. 3.**
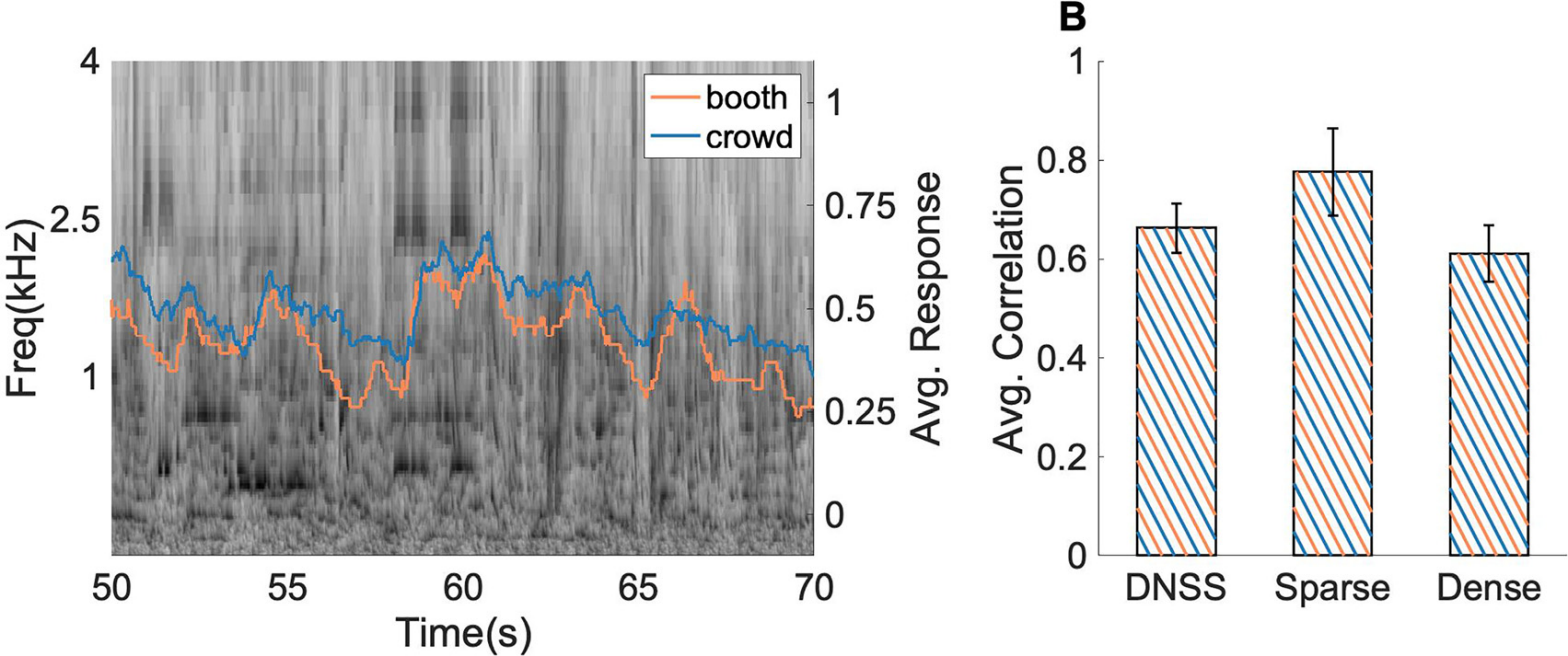
(Color online) Correlation between *booth* and *crowd* responses for DNSS scenes with an example. (A) Spectrogram of an example scene with *booth*, *crowd* average behavioral salience overlaid on the spectrogram. (B) Correlation between *booth* and *crowd* responses for DNSS scenes with *sparse* and *dense* breakup. Error bars depict ±1 SEM.

Next, salience responses across platforms from the *same* DNSS auditory scenes were compared. Correlations between average behavioral salience from *booth* and *crowd* revealed a strong agreement between the behavioral data under both testing paradigms (average Pearson correlation *ρ* = 0.69 across all scenes; p≪ 1e−3 for each DNSS scene). Figure 3(A) depicts a spectrogram of a sample scene overlaid on average behavioral salience from *booth* and *crowd* data in response to the same scene. While responses were rather noisy given the subjective and continuous behavioral feedback by subjects throughout the trial, there was a remarkable accord in general patterns between the two responses, revealing increased engagement of subjects with the scene at specific moments in time (regardless of the opposite scene or platform). Given the perceptual difference between scenes based on their acoustic transience, we further explored the correlation between *booth* and *crowd* average behavioral salience separately for dense and sparse DNSS scenes. Sparse scenes tended to give rise to more distinct objects and resulted in higher correlations (average correlation across scenes, *ρ* = 0.79), while denser scenes were more perceptually continual resulting in noisier (less distinct) reactions from subjects, though there was still statistical agreement in responses between *booth* and *crowd* data (average correlation across scenes, *ρ* = 0.64).

**FIG. 4.**
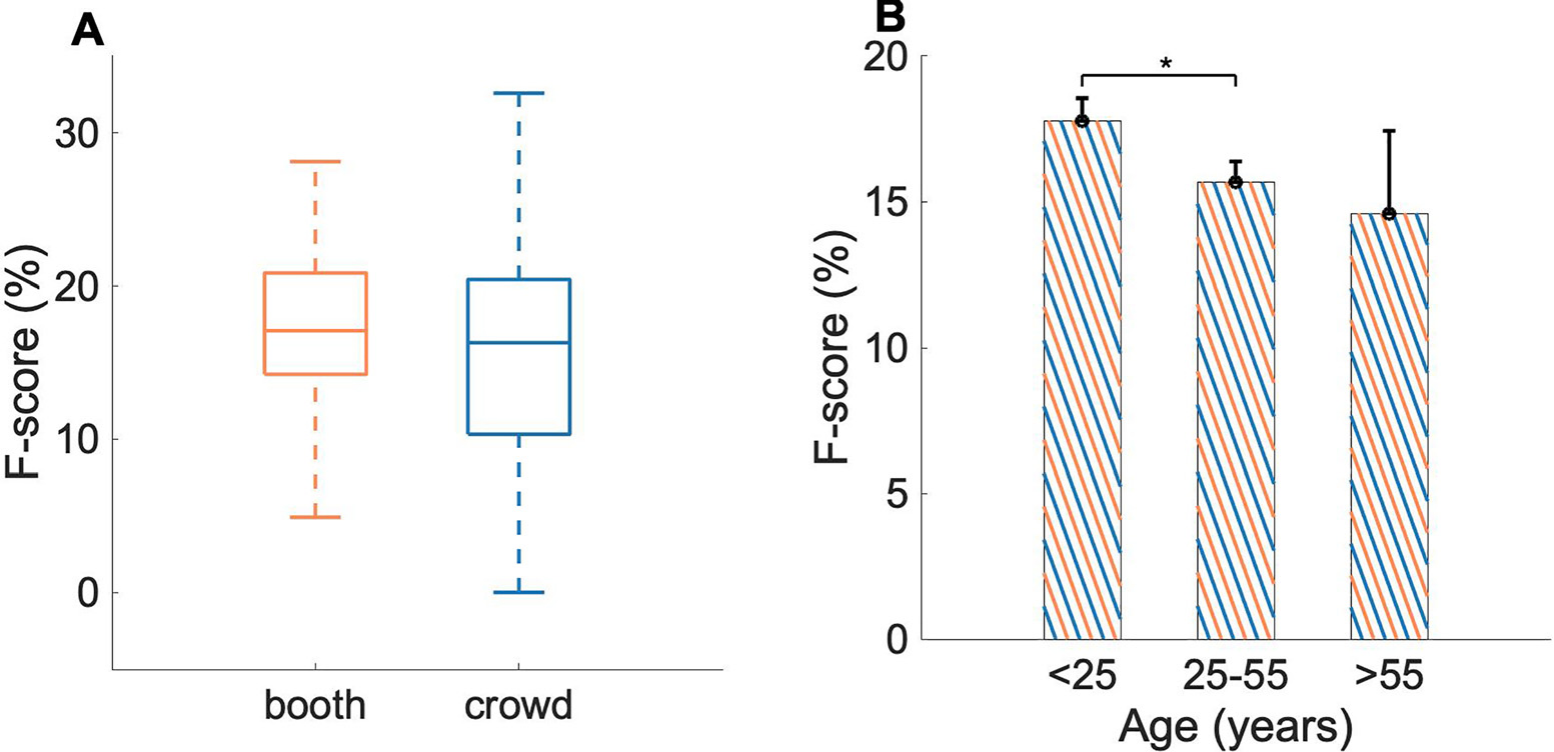
(Color online) Subjectwise F-scores indicating agreement of subjects with extracted events. Stars indicate significant differences between groups. Error bars depict ±1 SEM. (A) F-scores for *booth* and *crowd* subjects for matched scenes. (B) Combined F-scores from *booth* and *crowd* divided by age group.

In addition to agreement between *booth* and *crowd* data in average behavioral salience, inter-subject agreement based on salient event onsets also revealed a consistent concurrence across behavioral paradigms on average, albeit with higher variance for the crowd-sourced data. Subject-wise F-scores were computed as a measure of agreement between each subject's switching pattern and salient event onsets computed from the average behavioral salience for the same behavioral paradigm (*crowd* or *booth*, see Sec. II). A Kolmogorov-Smirnov test was used to confirm that the distributions were indeed normal since F-score is a harmonic mean of recall and precision (Massey, 1951). Subject-wise F-scores for *booth* (*p =* 0.99) and *crowd* (*p =* 0.93) using the DNSS scenes were found to follow a normal distribution. A two-sample t-test between subject-wise F-scores for *booth* and *crowd* showed no statistically significant difference between the two groups [*t*(136) = 1.30, *p =* 0.2]. Differences in variance in the F-scores for the two groups were also not found to be statistically significant [*F*(49, 87) = 0.6, *p =* 0.06] with higher variance observed in *crowd* (std = 0.07) when compared to *booth* (std = 0.05) [Fig. 4(A)].

To explore factors contributing to differences in salience responses between the *booth* and *crowd* paradigms, a three-way ANOVA was performed using age, platform, and gender as the factors where all factors were defined in a similar way as for reaction times. Although none of the factors or interactions were significant (Table II), gender and age seemed to have a stronger influence on the F-scores than platform, which suggests that the observed differences in F-scores were due to differences in populations and not due to differences in data collection platform. Effects of age on F-scores in the combined *booth* and *crowd* data are shown in Fig. 4(B). Subjects from *booth* and *crowd* were combined together and then grouped into three groups: young-age (<25 years), middle-age (25–55) and old-age (>55). The analysis showed no significant differences among age groups. Marginally significant differences were found between younger participants and middle age groups [two-sample t-test, *t*(128) = 1.94, *p =* 0.05] [Fig. 4(B)]; while no significant difference were observed between young and old groups [*t*(58) = 1.51, *p =* 0.13] and middle and older age groups [*t*(88) = 0.50, *p =* 0.62].

**FIG. 5.**
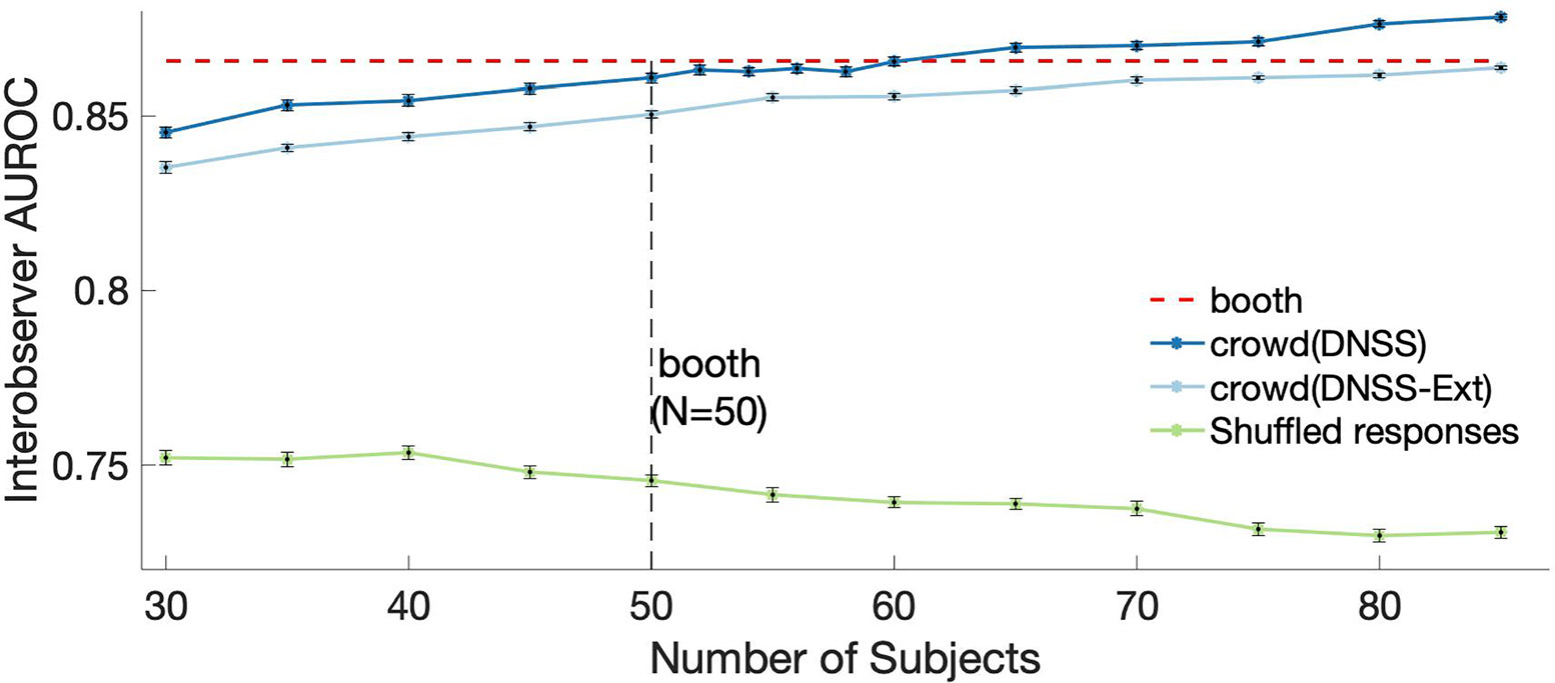
(Color online) Variability as function of number of subjects for the *crowd* data captured by interobserver ROC for *booth* (DNSS and DNSS-dense) and *crowd* (DNSS and DNSS-Ext scenes). Benchmark variability was calculated using data from all subjects in the *booth* condition. A chance-level interobserver AUROC computed from shuffled responses is shown for comparison purposes. Error bars represent standard errors across 50 trials of subsampling.

Next, we leveraged the crowd-sourced paradigm to examine the effect of subject quantity and used a measure of interobserver AUROC to quantify consistency across subjects as a function of the number of subjects (see Sec. II). Focusing on the DNSS scenes (for which there is a baseline in *booth* data), Fig. 5 shows that the interobserver AUROC increases systematically with the inclusion of more subjects, confirming increased consistency between subjects in terms of behavioral salience judgments. Furthermore, the analysis of the *crowd* data showed that reaching the level of consistency observed in *booth* data required *N =* 60 subjects, which is slightly more than *N =* 50 subjects used in the *booth* (Fig. 5, red line). This increasing trend in interobserver AUROC was also observed with DNSS-Ext scenes in *crowd* data, although the absolute values of AUROC were lower than for DNSS scenes. Chance level interobserver AUROC (Fig. 5, green line) was found to be lower than the *booth* and *crowd* interobserver AUROC and remained constant with increasing number of subjects. This difference supported our claim that a high interobserver AUROC indicated a high agreement across the subjects and the increase in AUROC was a systematic effect.

### B. Acoustic features

**FIG. 6.**
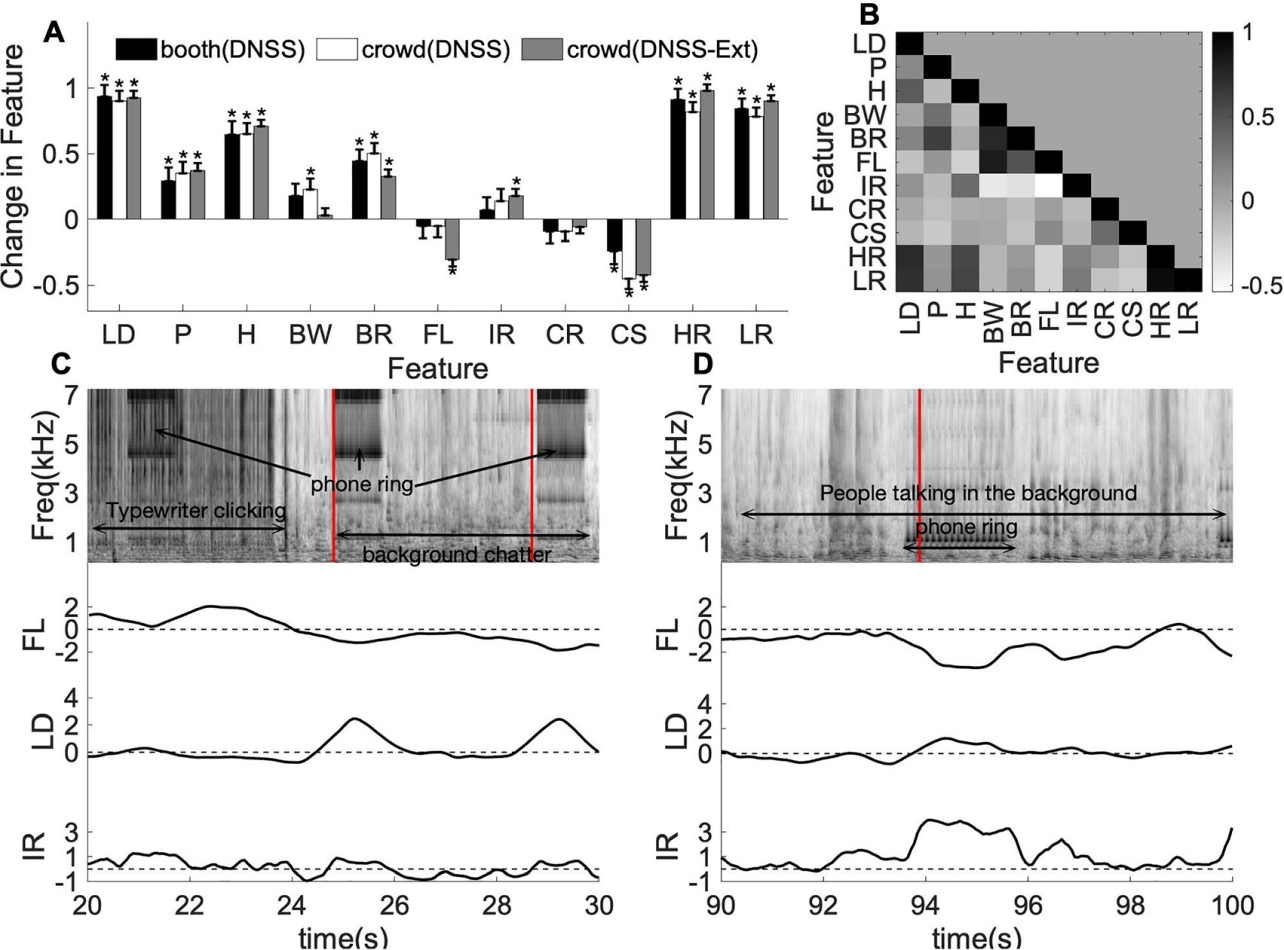
(Color online) (A) Acoustic feature change averaged across events, compared between sound *booth* and *crowd* for DNSS and DNSS-Ext scenes. All features are z-score normalized. Error bars represent ±1 standard error. (B) Correlations across features computed as Pearson correlation. (C) Example segment from one of the DNSS stimuli. (D) Example segment from one of the DNSS-Ext stimuli. (Top) Time-frequency spectrum of the signal, with a description of the scene in text and salient events marked by the vertical lines in red. Flatness, loudness, irregularity are shown in the bottom panels for the corresponding scenes.

Next, we examined the relationship between behavioral responses to salient events and changes in the acoustic structure of the scenes themselves. Figure 6(A) quantifies changes in eleven spectral and temporal acoustic dimensions (see Sec. II) around the salient events. In the first analysis, we compared acoustic changes around salient events in *booth* versus events in *crowd* data in the same DNSS scenes. Pair-wise two-sample t-tests for each acoustic dimension showed no significant differences between *booth* and *crowd* feature changes for DNSS across all features [bandwidth: *t*(418) = –0.4, *p =* 0.70, loudness: *t*(418) = 0.3, *p =* 0.76, pitch: *t*(418) = –0.5, *p =* 0.64, brightness: *t*(418) = –0.5, *p =* 0.62, harmonicity: *t*(418) = 0.0, *p =* 0.99, flatness: *t*(418) = –0.0, *p =* 0.99, irregularity: *t*(418) = –0.5, *p =* 0.61, rate: *t*(418) = –0.0, *p =* 0.99, scale: *t*(418) = 1.74, *p =* 0.09, high-rate: *t*(418) = 0.85, *p =* 0.40, low-rate: *t*(418) = 0.54, *p =* 0.59]. For each dataset, we noted that loudness, brightness, harmonicity, high-rate, and low-rate had significant positive changes while scale had significant negative changes around events [one-sample t-test, for *booth*, loudness: *t*(192) = 11.0, *p* = 6e−22, pitch: *t*(192) = 2.8, *p* = 0.01, brightness: *t*(192) = 4.8, *p*  =  1e−3, harmonicity: *t*(192) = 6.4, *p* = 1e−9, high-rate: *t*(192) = 11.0, *p*  =  4e−22, low-rate: *t*(192) = 10.5, *p* = 1e−20, scale: *t*(192) = –2.5, *p* = *0.01*, for *crowd*, loudness: *t*(226) = 11.8, *p*  =  3e−25, pitch: *t*(226) = 4.2, *p* =  4e−5, brightness: *t*(226) = 6.2, *p* =  3e−9, harmonicity: *t*(226) = 7.7, *p* =  4e−13, high-rate: *t*(226) = 11.1, *p* = 3e−22, low-rate: *t*(226) = 10.9, *p* = 1e−22, scale: *t*(226) = –3.9, *p* = 8e−4]. Bandwidth was the only feature which was found to have significant positive changes for the *crowd* events [*t*(226) = 2.7, *p* = 0.01], whereas for the *booth* events the changes in bandwidth were not statistically significant [*t*(192) = 1.9, *p* = 0.06]. These results suggested that subjects under both experimental paradigms (*booth* and *crowd*) were reacting to prominent variations along the same acoustic dimensions of the scenes, driven by loudness and mostly spectral attributes.

Taking advantage of the expanded variety of scenes tested in the *crowd* paradigm, we compared the effect of changes in acoustic attributes near salient onsets for DNSS-Ext scenes in the *crowd* data [Fig. 6(A)]. A one-sample t-test showed that loudness [*t*(554) = 16.9, *p* = 3e−52], pitch [*t*(554) = 6.2, *p* = 6e−10], brightness [*t*(554) = 5.9, *p* = 4e−9], harmonicity [*t*(554) = 14.0, *p* = 1e−38], irregularity [*t*(554) = 3.2, *p* = 1e−3], high-rate [*t*(554) = 20.4, *p* = 1e-69], low-rate [*t*(554) = 19.9, *p* = 2e−67] had significant positive changes while flatness [*t*(554) = –6.2, *p* = 1e−9] and scale [*t*(554) = –6.5, *p* = 2e−10] had significant negative changes around DNSS-Ext events. A two-sample t-test showed changes in bandwidth [*t*(780) = 2.03, *p* = 0.04] and flatness [*t*(780) = 2.9, *p* = 3e−3] were significantly different across DNSS and DNSS-Ext scenes.

It is important to note that the acoustic dimensions explored in this study are not necessarily independent of each other, and there exist strong correlations between several features as shown in Fig. 6(B). For example, loudness is strongly correlated to harmonicity (*ρ* = 0.44, *p* = 6e−3), high-rate (*ρ* = 0.73, *p* = 5e-48), and low-rate features (*ρ* = 0.69, *p* = 5e−70) while flatness is correlated to brightness (*ρ* = 0.49, *p* = 0.01).

The differences between DNSS and DNSS-Ext were largely driven by the variety of scene composition in each dataset. As noted earlier, the DNSS-Ext scenes consisted of a higher percentage of human vocalizations and device sounds. An example DNSS-Ext scene with a large change in flatness is shown in Fig. 6(D). This particular scene had a telephone ring event with some speech events in the background. A sharp dip in flatness can be observed when the ring happens due to the tone-like nature of the ring. This dip also coincided with an increase in loudness near t=25 s, as well as an increase in spectral irregularity [Fig. 6(D), bottom panels]. An example scene with a telephone ring from a DNSS scene is shown in Fig. 6(C) for comparison. The ring is of a different nature with a different frequency profile which caused a smaller dip in spectral flatness and not a pronounced change in irregularity [Fig. 6(C), bottom panels].

### C. Event prediction

With the consistency between *booth* and *crowd* data established with various characterizations based on behavioral data and acoustic changes, salience models derived from both platforms are expected to be compatible with each other. To confirm this, a salience computational model of event prediction based on acoustic changes was evaluated in a cross-platform manner (see Sec. II for model details). Models were trained separately on *booth* and *crowd* data for DNSS scenes and tested on held-out sets from both platforms using tenfold cross-validation. Table III shows the performance of the models, quantified using the AUROC. No significant differences were observed on paired t-tests on cross-validation folds for both models tested on *crowd* data [*t*(9) = –1.3, *p =* 0.33] and *booth* [*t*(9) = 1.0, *p* = 0.22].

**TABLE III.** Cross-modal AUROC averaged across tenfold cross-validation. Models were trained using DNSS scenes in *booth* and *crowd* data and tested on the held out sets from *booth* and *crowd* data from the same scenes. Standard error across 10 folds is indicated with ± sign.

	Test data	
Train data	*Booth*	*Crowd*
*Booth*	0.759 ± 0.027	0.753 ± 0.037
*Crowd*	0.753 ± 0.027	0.757 ± 0.038

**FIG. 7.**
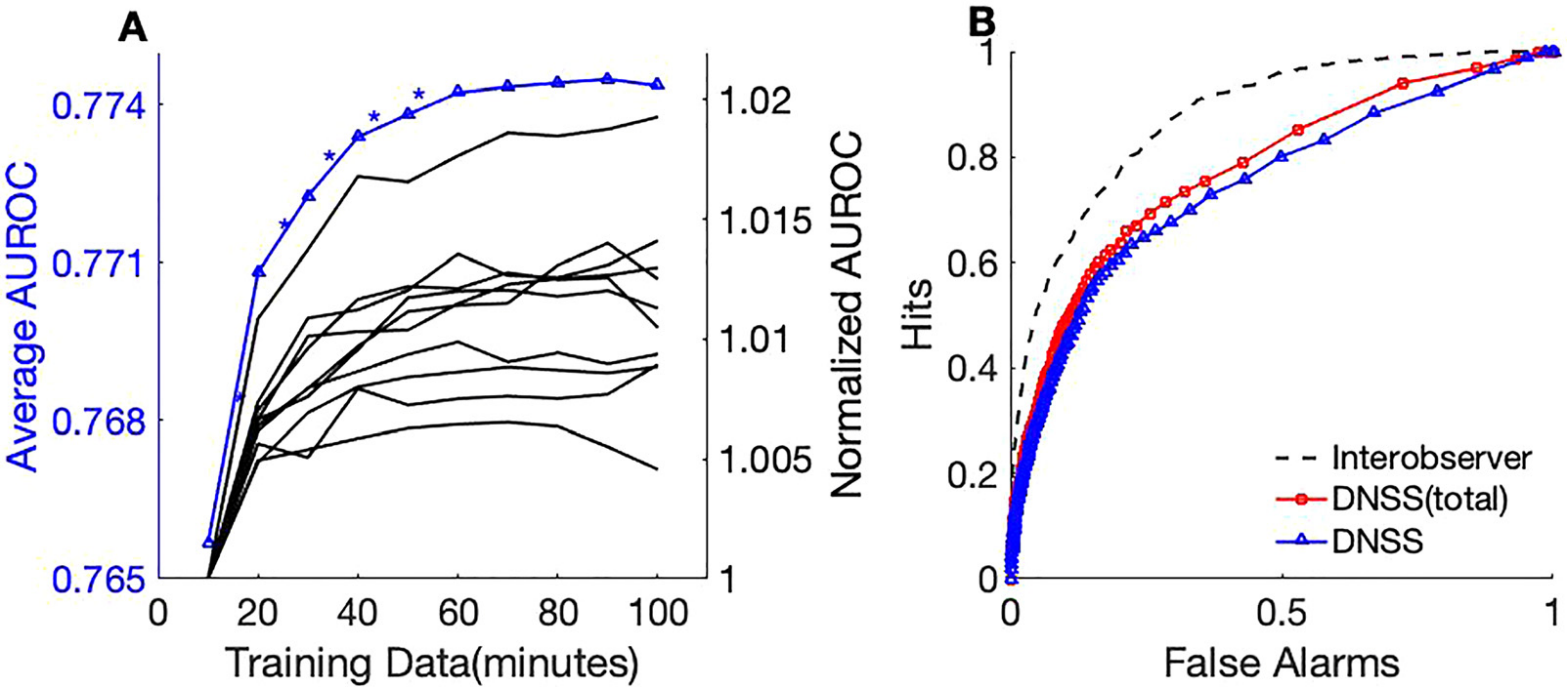
(Color online) (A) Event detection performance in terms of AUROC from incremental data augmentation. On the left *y* axis, the average AUROC of the 10-folds is plotted with each increment step and statistically significant improvements indicated with an asterisk. On the right *y* axis, each line represents a cross-validation fold and AUROC for each augmentation step is divided by the AUROC with 10 min of training data to obtain a normalized AUROC measure. All 56 scenes were used for this evaluation. (B) ROC with models trained on DNSS and DNSS-total when compared with the interobserver performance which can be considered an upper-bound to the detection performance.

With access to a larger scene dataset with a bigger pool of behavioral responses, we quantified the effect of additional data on acoustic predictions in an incremental manner. This analysis examined the hypothesis that computational salience models would benefit from larger databases (with more diverse scenes) that become possible with crowd-sourced methods. Using AUROC as a performance measure, Fig. 7(A) shows the effect of data augmentation with different amounts of training data on the detection performance. The evaluation is performed with 10-folds of test sets (11 min each) and for each test fold, training data is sampled from the remaining folds in steps of 10 min. Figure 7(A) (right *y* axis) shows a normalized AUROC for each fold in cross-validation, where the AUROC with 10 min of training data were used as the denominator. For each increment in the training data, a paired t-test was performed on the AUROC for the 10-folds to test if the performance improvement is significant. The analysis revealed a statistically significant increase ( p< 0.05) in performance with the inclusion of additional data up to 70 min [Fig. 7(A), left *y* axis] and the improvements were not statistically significant after 70 min. The model with 100 min improves AUROC by 1% relative to the 10 min model. Figure 7(B) shows the impact of the DNSS-Ext data on performance improvements with receiver operating characteristic (ROC) curves compared to a model trained on DNSS data only. Interobserver ROC serves as an upper limit on the achievable detection performance. As the figure indicates, higher amounts of data led to better detection performance.

## IV. DISCUSSION

In this study, we presented a detailed analysis of salience data for natural scenes, collected on a crowd-sourcing platform, using a dichotic listening paradigm. This work focused on three main goals: establishing the validity of dichotic listening in a crowd-sourcing platform as a reliable marker of auditory salience in complex natural scenes; expanding the existing salience dataset to more diverse scenes; and validating the benefits of large scale salience datasets to develop accurate salience models. As previously examined in several earlier studies, there is a close synergy between acoustic attributes of a sound stimulus and its perceptual salience. The acoustic analysis presented in Fig. 6(A) shows that key acoustic attributes like loudness, pitch, and harmonicity, which were previously shown to drive salience (Kaya and Elhilali, 2014; Tordini *et al.*, 2015; Tordini *et al.*, 2013), have statistically significant changes around event onsets from both the *booth* and *crowd* data. This result indicates that any large changes in these features induce a consistent attention shift in subjects even under dichotic listening. This observation is consistent with previous research in salience that hypothesized that violation of expectation or variations in time along key attributes of the signal give rise to salient events (Röer *et al.*, 2014; Vachon *et al.*, 2017). In the current work, these effects are confirmed in a larger and more diverse pool of subjects with the *crowd* data and provide further support for using dichotic listening for salience data collection. Moreover, the analysis presented here extends the investigation of acoustic attributes to include rate-specific energies (high rates HR and low rates LR) which are found to have significant changes around the salient events. The higher modulation energies (>20 Hz) represent roughness in the audio signal, and the contribution from high-rate towards salient events coincides with the known effects of roughness on salience (Arnal *et al.*, 2019). On the other end of the modulation spectrum, lower modulation energies (<20 Hz) are commensurate with dynamics in the audio signal (e.g., syllabic rate in speech) and play an important role not only in intelligibility and perception of natural sounds (Ding *et al.*, 2017; Elhilali, 2019; Elliott and Theunissen, 2009) but are found here to be important markers of auditory salience.

While changes in key acoustic attributes tend to draw the attentional focus of listeners, we note that variability and richness of everyday soundscapes do also result in differences of effects across different scene subsets, as noted with features such as flatness or irregularity. These variations could only be addressed with an expansion of scene datasets to an even larger size. Furthermore, the exploration of acoustic effects has to be carefully appraised as changes in acoustic attributes are heavily interdependent. As shown in Fig. 6(B), there are strong correlations across the feature dimensions, and the contribution of features to events is affected by these inter-dependencies. Earlier behavioral and neural recordings have shown a great deal of nonlinear interactions between features such as pitch and timbre (Allen and Oxenham, 2014; Melara and Marks, 1990; Walker *et al.*, 2011) that underlie the perception of integrated auditory objects. Such interdependence has also been reported in perceptual and neural measurements of auditory salience (Kaya *et al.*, 2020) where nonlinear interactions between acoustic attributes like pitch, intensity, and timbre cause interdependent responses to changes in individual dimensions. Given these multiplexed relationships, it is important to nuance judgments of which acoustic features are critical for salience perception and infer that no one feature can operate on its own as the main driver of sound conspicuity.

In line with our initial goal, the study establishes strong parallels between laboratory and online data. Similar to some of the crowd-sourced visual salience studies (Jiang *et al.*, 2015; Rudoy *et al.*, 2012), a comprehensive comparison of the data from *booth* and *crowd* sets using various metrics is performed to justify the adoption of the crowd-sourcing platform. Correlations of the average behavioral salience between *booth* and *crowd* data indicate considerable agreement across platforms [Fig. 3(B)]. Under the assumptions of normal distribution, this approach is similar to the divergence measures used in Rudoy *et al.* (2012). We also establish similarities across the *booth* and *crowd* platforms in terms of inter-subject variability. This variability is first quantified using subject-wise F-scores which measured variability within the platform around the salient events. We observe no statistically significant differences in average F-scores and variance in F-scores. Further breakdown of the differences in F-scores across reveals that age and gender are the main contributors to the small differences observed in *crowd* data (Table II). These are subject-specific factors that point to the diversity in the *crowd* data. The diversity of subjects guarantees an unbiased measure of salience, which is of utmost importance when developing datasets that can be used for salience models. In addition, interobserver agreement is used as a secondary measure of variability. As shown in Fig. 5, *crowd* data achieves the same interobserver AUROC as *booth* data with a slightly higher number of subjects and there is an increasing trend in AUROC with the number of subjects, which shows the necessity for a larger number of subjects to reduce variance in salience data. The analysis with interobserver agreement serves as validation of using a larger pool of subjects and can also be used as a guiding principle to choose the number of subjects for a required level of variance in data.

While it is relatively easier to collect crowd-sourced data from a large number of subjects, quality control is a concern that prevents the deployment for behavioral experiments (Brühlmann *et al.*, 2020; Kan and Drummey, 2018) on crowd-sourcing platforms. In this work, we use switching rates of subject responses as a quality measure of the subject data. A similar quality control strategy was used in BubbleView (Kim *et al.*, 2017), a visual exploration paradigm used for visual salience. Since the dichotic salience experiment inherently does not have a ground truth, it is difficult to inject control trials within the experiment, as is the case with some of the crowd-sourcing paradigms in visual salience (Othman *et al.*, 2017). While the switching rate analysis is done post-experiment in this study, it is possible to inform subjects when their switching rates fall outside the acceptable range. Another aspect related to the timing of subject response is the reaction times of the subjects. Although there are no significant differences in average reaction times across platforms [Fig. 2(D)], ANOVA on reaction times indicates that the age of the subjects can contribute (though not significant) more than the platform (Table II).

Advantages of having large amounts of data were demonstrated by several visual salience models that leverage deep learning methods (Jiang *et al.*, 2015; Kruthiventi *et al.*, 2017). These models were trained on crowd-sourced data and were found to generalize well to eye-fixation benchmarks. In recent years, there have been numerous studies that leveraged principles of auditory salience for audio event detection application (Kothinti *et al.*, 2019; Podwinska *et al.*, 2019). But none of these studies employed any behavioral data as part of the salience models, which could be partly attributed to the lack of large-scale salience data. In this work, we demonstrate the advantage of an extended auditory salience database; and consistent improvements from data augmentation support the rationale to scale the data collection over crowd-sourcing platforms. The performance of acoustic prediction is significantly improved by adding more data to the training set [Fig. 7(A)]. While these models are by no means comparable in size to deep learning models of visual salience, the improvement shown with added data is an encouraging outcome. There is a significant gap between the performance of the salience model and the inter-observer agreement which serves as an upper-bound on detection performance [Fig. 7(B)]. This difference could be because of the higher-level stimulus attributes such as scene semantics that could directly impact salience or modulate the importance given to different acoustic dimensions. We believe this gap could be bridged by studying a larger variety of scenes with complex semantics such as speech and music.

## APPENDIX

The details of the soundscapes used as part of the study are listed in Table IV.

**TABLE IV.** Details of the soundscapes used in the study.

#	Block	Duration	Description	Sparse	Source
1	DNSS	2:00	Nature, Birds	No	Youtube
2	DNSS	2:15	Sporting Event	Yes	BBC Sounds
3	DNSS	2:00	Cafeteria	No	BBC Sounds
4	DNSS	2:02	Battle, Guns	Yes	Youtube
5	DNSS	1:21	Airplane, Baby	Yes	Soundsnap
6	DNSS	1:13	Fair	No	Freesound
7	DNSS	1:59	Classical Music	No	Youtube
8	DNSS	2:01	Bowling Alley	Yes	BBC Sounds
9	DNSS	2:00	Egypt Protests	No	Youtube
10	DNSS	2:08	Store Counter	No	BBC Sounds
11	DNSS	1:57	Drum Line	No	Youtube
12	DNSS	1:59	Blacksmith	No	Youtube
13	DNSS	2:13	Orchestra Tuning	No	Youtube
14	DNSS	2:07	Japanese Game Show	No	Youtube
15	DNSS	2:22	Dog Park	No	Freesound
16	DNSS	2:14	Casino	No	Freesound
17	DNSS-Ext	2:00	Carnival, crowds	No	Freesound
18	DNSS-Ext	2:00	Arabic Concert	No	Freesound
19	DNSS-Ext	2:00	Cafeteria, Vehicles	No	Freesound
20	DNSS-Ext	2:00	Guns, Heavy Artillery	No	Freesound
21	DNSS-Ext	2:00	Baby Laughing	No	Freesound
22	DNSS-Ext	2:00	Beach, Waves	No	Freesound
23	DNSS-Ext	2:00	Dogs Growling, Birds	No	Freesound
24	DNSS-Ext	2:00	Crowd Chanting, Clapping	No	Freesound
25	DNSS-Ext	2:00	Volleyball Game, Cheering	No	Freesound
26	DNSS-Ext	2:00	Social Gathering, Dishes	No	Freesound
27	DNSS-Ext	2:00	Birds, Insects Chirping	No	Freesound
28	DNSS-Ext	2:00	Rain, Thunders	No	Freesound
29	DNSS-Ext	2:00	Piano Concert	No	Freesound
30	DNSS-Ext	2:00	Goats, Dogs, Horses	No	Freesound
31	DNSS-Ext	2:00	Water Stream, Birds	No	Freesound
32	DNSS-Ext	2:00	Party, Laughing, Music	No	Freesound
33	DNSS-Ext	2:00	Arcade, Songs	No	Freesound
34	DNSS-Ext	2:00	Reception Area, Telephone	No	Freesound
35	DNSS-Ext	2:00	Parade, Cheering	No	Freesound
36	DNSS-Ext	2:00	Motorcycle, Pump	No	Freesound
37	DNSS-Ext	2:00	Crowd Fight, Sword Sounds	No	Freesound
38	DNSS-Ext	2:00	Carpentry Machinery	No	Freesound
39	DNSS-Ext	2:00	Motorbikes, Rain	No	Freesound
40	DNSS-Ext	2:00	Sports Arena, Music	No	Freesound
41	DNSS-Ext	2:00	Ocean Waves	No	Freesound
42	DNSS-Ext	2:00	String Instrument	No	Freesound
43	DNSS-Ext	2:00	Arcade, Gaming	No	Freesound
44	DNSS-Ext	2:00	Street, Drums	No	Freesound
45	DNSS-Ext	2:00	Sports Arena, Cheering	No	Freesound
46	DNSS-Ext	2:00	Market, Crowd	No	Freesound
47	DNSS-Ext	2:00	Restaurant, Dishes	No	Freesound
48	DNSS-Ext	2:00	Office Space, Telephone	No	Freesound
49	DNSS-Ext	2:00	Seagulls, Motorboat,	No	Freesound
50	DNSS-Ext	2:00	Piano Concerto	No	Freesound
51	DNSS-Ext	2:00	Birds	No	Freesound
52	DNSS-Ext	2:00	Birds, Rooster	No	Freesound
53	DNSS-Ext	2:00	Fireworks	No	Freesound
54	DNSS-Ext	2:00	Bar, Music	No	Freesound
55	DNSS-Ext	2:00	Birds, Crickets	No	Freesound
56	DNSS-Ext	2:00	Soccer Game	No	Freesound
